# Local eosinophils are associated with increased IgA subclass levels in the sinonasal mucosa of chronic rhinosinusitis with polyp patients

**DOI:** 10.1186/s13223-020-00428-y

**Published:** 2020-04-25

**Authors:** Hossein Aazami, Farhad Seif, Babak Ghalehbaghi, Pegah Babaheidarian, Alireza Mohebbi, Aslan Ahmadi, Majid Khoshmirsafa, Sahand Ghalehbaghi, Babak Behnam, Kobra Zinat Entezami, Zahra Madjd, Reza Falak

**Affiliations:** 1 Department of Immunology, School of Medicine, Iran University of Medical Sciences, Tehran, Iran; 2 Department of Immunology and Allergy, Academic Center for Education, Culture and Research, Tehran, Iran; 3 Neuroscience Research Center, Iran University of Medical Sciences, Tehran, Iran; 4 ENT and Head and Neck Research Center and Department, Hazrat Rasoul Akram Hospital, The Five Senses Institute, Iran University of Medical Sciences, Tehran, Iran; 5 Department of Pathology, Rasoul Akram Medical Complex, Iran University of Medical Sciences, Tehran, Iran; 6 Immunology Research Center, Institute of Immunology and Infectious Diseases, Iran University of Medical Sciences, Tehran, Iran; 7 Department of Medical Genetics and Molecular Biology, Faculty of Medicine, Iran University of Medical Sciences, Tehran, Iran; 8 Oncopathology Research Center, Iran University of Medical Sciences, Tehran, Iran

**Keywords:** CRSsNP, CRSwNP, B cell, IgA, Class switching, Nasal polyps, Chronic rhinosinusitis

## Abstract

**Background:**

Chronic rhinosinusitis (CRS) describes an inflammatory condition affecting the sinonasal mucosa. As the immune system players such as immunoglobulins play prominent roles in the development of CRS, we aimed to investigate the expression of IgA subclasses and factors involved in IgA class switching in the sinonasal mucosa of CRS patients.

**Methods:**

Specimens were collected from the sinonasal mucosa of the healthy controls and CRS patients. Histological assessments were performed by H&E and immunohistochemistry. Real-time PCR and ELISA methods were applied to measure gene expression and protein levels extracted from tissue samples, respectively.

**Results:**

We observed that total IgA and subclass-positive cells were higher in the patient groups than controls. There was a significant correlation between the number of eosinophils and total IgA and subclasses-positive cells (Pv < 0.0001). The expression of CXCL13, BAFF, AID, and germline transcripts were increased in CRSwNP patients. In contrast to IgA2 levels, IgA1 levels were significantly increased in the sinonasal tissue of CRSwNP patients (Pv < 0.01). TGF-β was significantly elevated in the sinonasal tissue of patients with CRSsNP.

**Conclusions:**

Increased protein levels of IgA subclasses and related antibody-producing cells were associated with elevated eosinophils in CRSwNP patients which may result in eosinophil pathological functions. Several therapeutic approaches might be developed to modulate the IgA production to ameliorate the inflammatory mechanisms in CRSwNP patients.
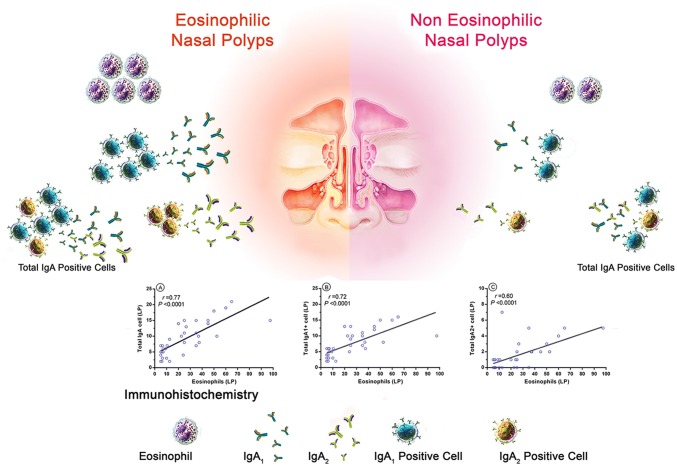

## Background

Chronic rhinosinusitis (CRS) refers to a chronic condition associated with local inflammation of sinonasal airways [1]. Based on the signs and symptoms, CT and endoscopic criteria, CRS is divided into two main groups, including CRS with nasal polyps (CRSwNP) and CRS without nasal polyps (CRSsNP) [2, 3]. Although the exact etiology of CRS remains unknown, immunological mechanisms are suspects in its initiation and progression.

Immunoglobulins (Igs) are major players of humoral immunity against infectious agents. The mucosa of sinonasal tracts encounters various microbes; therefore, IgA may play a protective role in response to sinonasal pathogenic microbes [4, 5]. IgA is the main secretory antibody found in mucosal surfaces and fluids which is produced by mucosal plasma cells and is translocated to the tracts of mucosal sites [6]. IgA1 and IgA2, as the most important isotypes of IgA in human beings, display distinct expression patterns in different mucosal tissues. IgA1 is dominantly expressed in airway tracts whereas IgA2 is predominantly found in secretions of the large intestine. However, both isotypes are approximately expressed in equal concentrations in the small intestine [7].

IgA is produced by B cells upon cytokine- and chemokine-stimulation provided by local inflammatory cells [8]. In this regard, some factors such as B cell–Activating Factor of the TNF family (BAFF, also named BLyS) and A Proliferation Inducing Ligand (APRIL) play pivotal roles in the maturation as well as the survival of B cells and plasma cells [9]. Moreover, they promote IgA class switching in a T cell-independent manner [10, 11].

Conversely, TGF-β is another cytokine produced by a variety of cell types, including fibroblasts and helper T cells. In fact, it is involved in T cell-dependent IgA class switching by regulation of germline transcripts [12–14]. In other words, Iα1-Cα1 and Iα2-Cα2 germline transcripts are produced by transcription of un-rearranged constant heavy chain (CH) genes through class switching to IgA subclasses [15]. Class switching occurs at constant regions of antibodies and is mainly performed by Activation Induced Cytidine Deaminase (AID) enzyme in activated B cells [16]. AID is normally expressed in germinal center B cells of lymphoid organs and mucosal tissues. Some distinct sequences of DNA, named as switch (S) regions, are located at the upstream of the Ig isotype genes (except for IgD). Upon the proximity of donor and acceptor in S region, DNA is broken and the formerly rearranged V domains of Ig heavy-chains are expressed in association with a different C region instead of initial Cµ, thus class switching to IgA, IgG, or IgE isotypes occur [17].

Another aspect of class switching is comprised of circular DNA deletion, so-called Switch Circles (SC), from the Ig heavy-chain loci through recombination. Switch circles consist of the DNA composition between the Sµ and a downstream S region as well as Intervening (I) region promoter located at the upstream of Sµ. Under the control of the I promoter, the SC produces an Iα–Cµ transcript, named circle transcript which contributes to AID in order to show ongoing class switching [18]. However, the process of local class switching to IgA subclasses is not fully determined in CRS patients. Therefore, we aimed to investigate the expression of IgA subclasses and also factors involved in the promotion of IgA class switching in the sinonasal mucosa of CRS patients.

## Methods

### Patients and controls

Patients suffering from CRS were recruited from the ENT, head, and neck clinic at Rasoul Akram Hospital, IUMS. A total of 36 CRSwNP patients and 12 CRSsNP patients took part in the study. Control inferior turbinate tissues (n = 22) were collected from subjects undergoing rhino-septoplasty as a result of septum deviation without a history of asthma or CRS. Demographic data of all participants are listed in Table 1. Nasal polyps was diagnosed based on the endoscopy and CT scan results, corresponding to the European Position Paper on Rhinosinusitis and Nasal Polyps [19]. This study was approved by the ethics committee of IUMS (IR.IUMS.FMD.REC1394.9211127203) and all participants filled written informed consent for tissue sample gathering. CRS patients who had immunodeficiencies, cystic fibrosis, bronchiectasis, chronic obstructive pulmonary disease, diabe-tes mellitus, neoplasia, fungal rhinosinusitis, upper airway infections within 1 month were excluded from the study. None of the participants were preg-nant or have been lactating; or used systemic or local corticosteroids, antihistamines, decongestants, antibiotics, and anti-leukotrienes within 4 weeks prior to surgery.

**Table 1:** Characteristics of CRS patients and controls

Characteristics	Controls	CRSsNP	CRSwNP
Number	22	12	36
Females/males	8/14	4/8	12/24
Age (y), median (IQR)	28 (24.7–33)	28 (17.2–36)	34.5 (30–41.7)
Asthma	–	–	8
CT score	–	7.5±5	14.3±6.6
Endoscopic score	–	0.7±0.6	1.9±0.7
VAS score	–	5.6±2.5	8.9±3

*CT Score* Computed tomography score, *IQR* Interquartile range, *CRSsNP* Chronic rhinosinusitis without nasal polyps, *CRSwNP* Chronic rhinosinusitis with nasal polyps, *VAS score* Visual analogue scale score

### Biopsies and specimens

Tissue samples were obtained from ethmoid mucosa and cut into three pieces; two sections were immediately stored at −80 °C for RNA and protein extractions. The third section was fixed overnight in a freshly prepared fixative containing 4% paraformaldehyde in PBS (pH 7.4) and finally embedded in paraffin wax for immunohistochemistry (IHC) tests.

### Histologic analysis

Tissue slides were prepared from the paraffin-embedded tissues and subsequently 3 μm sections stained by Hematoxylin & Eosin (H&E) to study the pathologic features of the samples. The frequency of eosinophils, neutrophils, mononuclear cells, total inflammatory cells, goblet cells, and mucosal glands was determined using an Olympus CX-40 light microscope (Olympus, Tokyo, Japan) with high power field (HPF:400X) and 5 random HPFs were evaluated by two independent pathologists who were blind to the clinical information. The data were presented as cells or glands per HPF. We also divided CRSwNP patients into two subgroups, one subgroup was defined as eosinophilic when eosinophils comprised more than 10% of the total inflammatory cells–as the cut-off–and another subgroup was defined as non-eosinophilic when eosinophils were less than 10% of the total inflammatory cells [20].

### Immunohistochemistry

In brief, sinonasal tissues were dehydrated and embedded in the paraffin and sectioned at 3 µm diameters. After rehydration and blocking of the endogenous peroxidase activity with 3% H_2_O_2_/methanol, the sections were washed with Tris-buffered saline (TBS) and blocked (with PBS, pH 7.4, containing 2% bovine serum albumin (Sigma-Aldrich, Darmstadt, Germany), 0.1% Triton X-100, and 0.1% sodium azide) at room temperature (RT) to reduce nonspecific bindings for 30 min to hinder nonspecific binding [21]. Then, the sections were incubated with an appropriate concentration of the antibodies for 1 h at RT. The details of which are as follows: primary antibodies, including polyclonal rabbit anti-human IgA antibody (at 1:100 dilution; Abcam, Cambridge, MA, USA), anti-human IgA1 antibody (at 1:200 dilution; ab193187), anti-human IgA2 antibody (1:100; ab193169, Abcam), monoclonal mouse anti-human CD20 (at 1:200 dilution; clone L26, Dako, Glostrup, Denmark), anti-human CD138 (1:100; Clone M115) were applied. After 2 h incubation, the slides were washed with TBS for 10 min and again incubated for 45 min at 30 °C with EnVision™ (Dako). The samples were coun-terstained with Mayer’s hematoxylin stain and mounted in Faramount Mounting Medium (Dako), before microscopic examination.

### Quantitative real-time polymerase chain reaction

Total RNA was isolated from nasal tissues with Trizol (Invitrogen, USA) according to the manufacturer’s instructions and the integrity of RNA was controlled by electrophoresis on 2% denaturing agarose gel. The minor genomic DNA contaminations were then removed by RNase-free DNase Set (Qiagen, Chatsworth, CA, USA) and 500 ng of total RNA from each sample was subjected to first-strand cDNA synthesis using RevertAid™ First Strand cDNA Synthesis Kit (MBI, Fermentas, USA). The success of the reverse transcription reaction was monitored by PCR amplification of glyceraldehyde-3-phosphate dehydrogenase transcripts. Real-time PCR reactions were carried out in total 20 µL volumes in Rotor-Gene Q machine (Qiagen, Hilden, Germany), using 10 µL of 2 × SYBR Green Master Mix (Takara), 1 µL of cDNA, and 1 µL of 200 nM mixture of forward and reverse primers in duplicate. The primer sequences are listed in Table 2. The temperature profile included 40 PCR cycles with 95 °C denaturation for 5 s and 60 °C annealing and extension for 30 s. The mean threshold cycle values were normalized to the expression of beta-actin (β-actin) and the relative mRNA expression levels of target genes were calculated using 2^−ΔΔCt^ method.

**Table 2:** Primers used for quantitative PCR

Primers	Forward and reverse sequences
μ GLT	F: 5’-GTGATTAAGGAGAAACACTTTGAT-3’ R: 5’-AGACGAGGGGGAAAAGGGTT-3’
α1 GLT	F: 5’-CTCAGCACTGCGGGCCCTCCA-3’ R: 5’-GTTCCCATCTGGCTGGGTGCTGCA-3’
α2 GLT	F: 5’-CTCAGCACTGCGGGCCCTCCA-3’ R: 5’-GTTCCCATCTTGGGGGGTGCTGTC-3’
Iα-Cμ	F: 5’-CAGCAGCCCTCTTGGCAGGCAGCCAG-3’ R: 5’-AGACGAGGGGGAAAAGGGTT-3’
AID	F: 5’-CACCACTATGGACAGCCTCTTG-3’ R: 5’-ACTGTCACGCCTCTTCACTACG-3’
BAFF	5’-TGCCTGAAACACTACCCAATAA-3’ R: 5’-AGCAGTTTCAATGCACCAAA-3’
APRIL	F: 5’-GCCAGGTCCTGTTTCAAGACGT-3’ R: 5’-TGTAAATGGAAGACACCTGCGC-3’
CXCL13	F: 5’-GCTTGAGGTGTAGATGTGTCC-3’ R: 5’-CCCACGGGGCAAGATTTGAA-3’
β-actin	F: 5’-TCCCTGGAGAAGAGCTACG-3’ R: 5’-GTAGTTTCGTGGATGCCACA-3’

### Measurement of immunoglobulins and cytokines

The biopsies were weighed and homogenized in a pre-cooled pestle and mortar with an appropriate amount of ice-cold PBS supplemented with 0.05% Tween 20 (Sigma-Aldrich, St Louis, Mo, USA) and 1% cocktail of protease inhibitors (Santa Cruz, California, USA). In brief, 1 μL of buffer added per 1 mg of the tissue, upon homogenization the content was centrifuged at 4000*g* for 20 min at 4 °C. Supernatants were aliquoted and stored at −80 °C. Total IgA was measured by Minineph™ human IgA kit (ZK010.R, Birmingham, UK) using Minieph nephelometry instrumentation (Binding Site, UK). We used sandwich ELISA kits for measurement of IgA subclasses, including IgA1 and IgA2 (MyBioSource, San Diego, USA) and for determination of the active form of TGF-β1 (R&D Systems, Minneapolis, USA) according to manufacturers’ instructions The minimum detectable amount of the applied kits was 28 ng/ml, 4 ng/ml, and 31 pg/ml for IgA1, IgA2, and TGF-β, respectively. The total protein of the extracts was measured with BCA method (Bio-Rad, Hercules, California) and the concentrations of total IgA, IgA1, IgA2, and TGF-β in the lysates were normalized to the protein concentrations.

### Statistical analysis

Statistical analysis was performed with SPSS version 24.0 (SPSS Inc., Chicago, Illinois, USA) and plots were depicted by Prism version 6.1 software (GraphPad, La Jolla, California). Kolmogorov–Smirnov test was used to evaluate the normal distribution of the data. Data were expressed as medians and interquartile ranges (IQR) unless otherwise noted, and statistical significance was analyzed using nonparametric Mann–Whitney U test to determine intergroup differences and Kruskal–Wallis H for evaluation of the differences between groups. The Spearman test was used to determine correlations between cellular frequencies. P-values less than 0.05 were considered statistically significant.

## Results

In this study 22 controls (8 females and 14 males), 12 CRSsNP patients (4 females and 8 males) and 36 CRSwNP patients (12 females and 24 males) participated. The median (IQR) age of the participants was 28 (24.7–33), 28 (17.2–36) and 34.5 (30–41.7), respectively. The comparison of the endoscopic score, CT score, and VAS score of the CRS patients with controls are summarized in Table 1.

### The number of inflammatory cells was increased in CRS patients

**Fig. 1:**
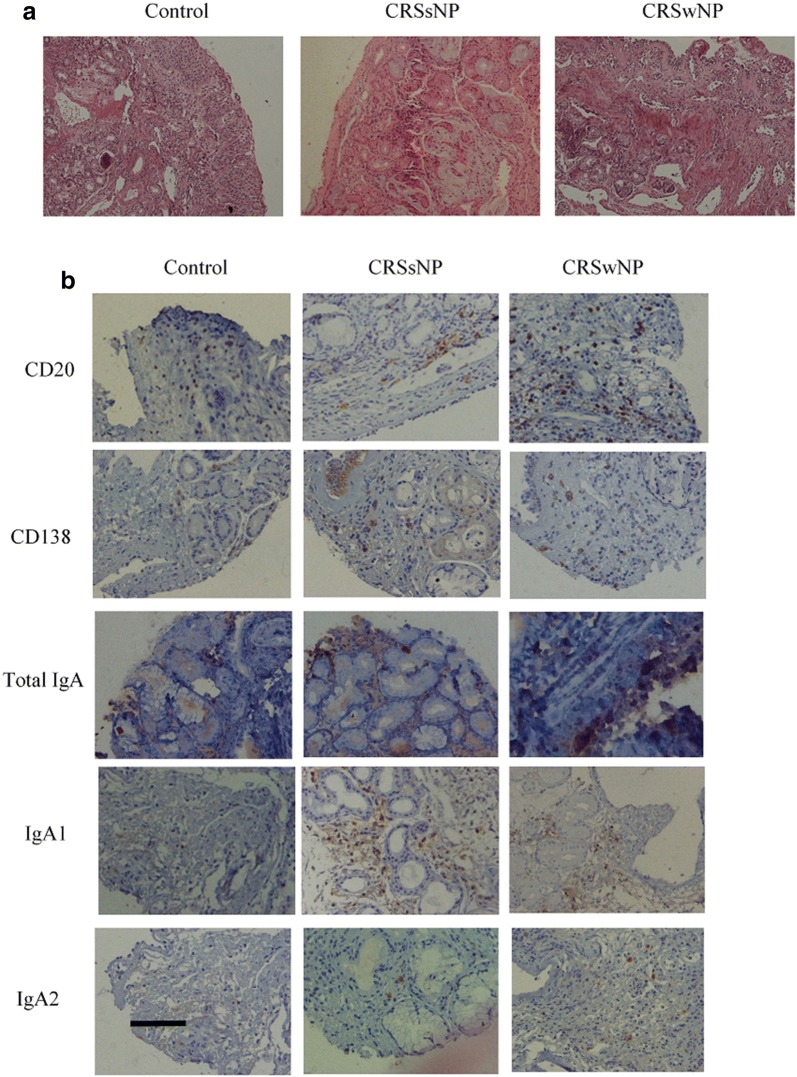
The inflammatory pattern of sinonasal tissues. **a** H&E staining and **b** IHC staining related to CD20^+^ (representing B cells), CD138^+^ (representing plasma cells), total IgA^+^, IgA1^+^, and IgA2^+^ cells (indicating IgA producing cells); in healthy controls and CRS patients. Scale bar 100 µm. *CRSsNP* Chronic rhinosinusitis without nasal polyps, *CRSwNP* Chronic rhinosinusitis with nasal polyps

Evaluation of H&E stained slides showed that CRS patients had higher eosinophils than controls (*P *< 0.001). There was no significant difference between the eosinophil count of CRSsNP and CRSwNP groups (Fig. 1 and Table 3). The frequency of neutrophils was significantly higher in CRSwNP and CRSsNP groups than controls (*P *< 0.001 and *P *= 0.023, respectively). There was no significant difference between neutrophil count of CRSsNP and CRSwNP groups (Table 3). The number of total inflammatory cells was significantly higher in both CRS groups and there was also a significant difference between CRSwNP and CRSsNP groups (*P *< 0.001 for CRSwNP vs controls, *P *= 0.031 for CRSsNP vs controls and *P *= 0.002 for CRSsNP vs CRSwNP).

**Table 3:** General histologic results of control, CRSsNP, and CRSwNP groups

	Control	CRSsNP	CRSwNP	Control vs. CRSsNP	Control vs. CRSwNP	CRSwNP vs. CRSsNP
Neutrophils	0.5 (0.5–1.5)	2 (1–4.5)	5 (2.5–9.25)	*0.023*	*<0.001*	0.85
Eosinophils	0.5 (0.5–1)	11.5 (4.5–17.5)	23.25 (6.75–36)	*< 0.001*	*< 0.001*	0.1
Mononuclear cells	9.5 (6–13)	13.5 (8.5–19)	26 (15–35)	0.275	*< 0.001*	*0.007*
Goblet cells	13.5 (10.5–19)	12.5 (10–15.5)	7.5 (5–14.25)	0.078	*0.008*	0.71
Mucosal gland	22.5 (17.5–32)	15.5 (10–20)	13.5 (7.5–27.5)	0.702	*0.013*	*0.005*
Inflammatory cells	12 (8.5–18.5)	29.75 (17.25–43)	63.75 (48–88.75)	*0.031*	*< 0.001*	*0.002*

Values are expressed as medians (IQR). The Mann-Whitney U test was used for unpaired comparisons

*CRSsNP* Chronic rhinosinusitis without nasal polyps, *CRSwNP* Chronic rhinosinusitis with nasal polyps

*P* < 0.05 was considered statistically significant. Italics P values show significant results (<0.05)

### The number of eosinophils correlated with IgA producing cells

**Fig. 2:**
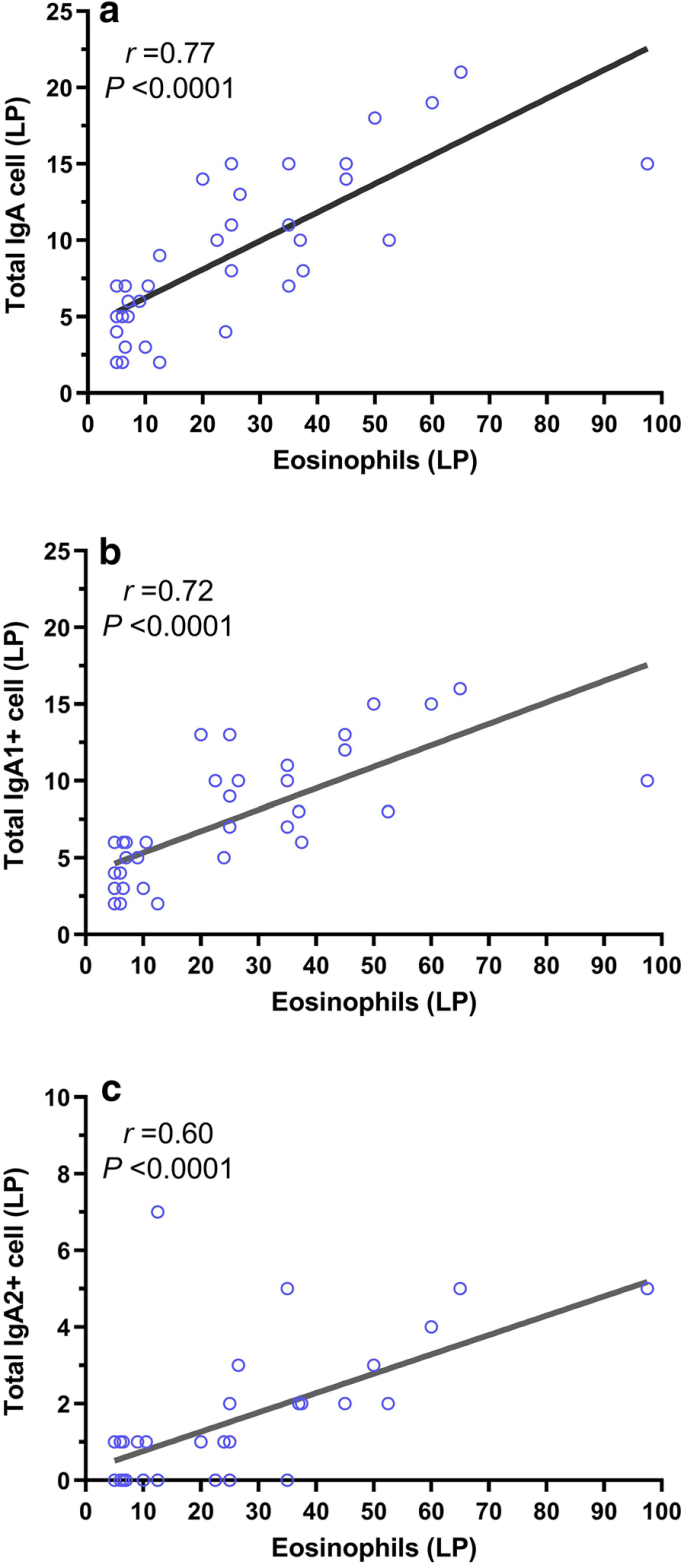
The number of eosinophils showed positive significant correlations with the total IgA + cells (r = 0.77) (**a**), IgA1 + cells (r = 0.72) (**b**), and IgA2 + cells (r = 0.6) (**c**) in lamina propria (LP) of CRSwNP patients according to Spearman’s rank correlation test

The number of CD20^+^ cells (a marker of naive B cells) was elevated in CRS patients in comparison to controls (*P *< 0.001 and *P *= 0.001, respectively), but there was no significant difference between CRSsNP and CRSwNP groups. The frequency of CD138^+^ cells (a marker of plasma cells) was increased in CRSwNP patients in comparison to CRSsNP and controls (*P *= 0.009 and *P *< 0.001, respectively). Total IgA^+^ cells were elevated in the lamina propria (LP) of both CRS patients compared with controls (*P *< 0.001). However, the evaluation of total IgA^+^ cells in the epithelium (EP), only revealed a difference in the CRSwNP patients and controls (*P *= 0.002). The number of IgA1^+^ and IgA2^+^ cells in the LP and IgA1^+^ cells in the EP of CRS patients was higher than the controls. In contrast, there was no significant difference in the frequency of IgA2^+^ cells in the EP of CRSwNP and CRSsNP groups (Table 4). Notably, the number of infiltrating eosinophils significantly correlated with the total IgA expressing cells and also IgA subtypes expressing cells in LP of CRSwNP patients (P < 0.0001) (Fig. 2).

**Table 4:** Immunohistochemistry results of control, CRSsNP and CRSwNP groups

	Control	CRSsNP	CRSwNP	Control vs. CRSsNP	Control vs. CRSwNP	CRSwNP vs. CRSsNP
Total IgA LP	3 (2–4)	6.5 (5–8.5)	8 (5–13.5)	*< 0.001*	*< 0.001*	0.77
IgA2 LP	0 (0–0)	1 (1–1)	1 (0–2)	*0.023*	*< 0.001*	0.721
IgA1 LP	2.5 (2–3)	6.5 (5–8.5)	6.5 (4–10)	*<0.001*	*<0.001*	0.82
Total IgA EP	1 (1–1)	1 (0.5–2)	2 (1–3)	0.34	*0.002*	0.122
IgA2 EP	0 (0–0)	0 (0–0.5)	0 (0–1)	0.1	0.11	0.1
IgA1 EP	1 (1–1)	1 (0.5–2)	2 (1–2)	0.34	*0.001*	0.11
CD20	1 (1–2)	5.5 (3–8)	14 (6.5–34.5)	*0.001*	*< 0.001*	0.134
CD138	4 (2–5)	5 (2–7)	10.5 (5–33.5)	0.327	*< 0.001*	*0.009*

Values are expressed as medians (IQR). Mann-Whitney U test was used for unpaired comparisons

*CRSsNP* Chronic rhinosinusitis without nasal polyps, *CRSwNP* Chronic rhinosinusitis with nasal polyps, *LP* Lamina propria, *EP* Epithelium

P < 0.05 was considered statistically significant. Italic P values show significant results (<0.05)

### IgA positive cells were increased in ENP subgroup

According to the proportion of eosinophils to total inflammatory cells [20], we divided CRSwNP patients into 23 eosinophilic CRSwNP (ENP) and 13 non-eosinophilic CRSwNP (N-ENP) subgroups. ENP patients had higher neutrophils than N-ENP and we observed only a significant difference between neutrophil count of ENP and CRSsNP patients (*P *= 0.005). We found that total inflammatory cells in the ENP patients were significantly higher than N-ENP patients (Table 5). The number of CD20^+^ cells was significantly increased in the sinonasal mucosa of ENP in comparison to CRSsNP patients (*P *= 0.026). In addition, the frequency of plasma cells was significantly elevated in the ENP subgroup compared with N-ENP and CRSsNP patients (*P *= 0.022 and *P *= 0.01, respectively). Total IgA^+^ cells were elevated in the LP and EP of ENP subgroup compared with N-ENP and CRSsNP patients. The number of IgA1^+^ cells in the LP and IgA2^+^ cells in the EP was enhanced in ENP patients compared with N-ENP patients. The frequency of IgA2^+^ cells in the LP of ENP patients was increased in comparison to N-ENP and CRSsNP patients. There was no significant difference between the number of IgA1^+^ cells of EP among ENP, N-ENP, and CRSsNP patients (Table 6).

**Table 5:** General histologic results of eosinophilic and non-eosinophilic CRSwNP subgroups

	ENP	N-ENP	ENP vs. N-ENP	ENP vs. CRSsNP	N-ENP vs. CRSsNP
Neutrophils	5 (2.5–9.75)	3.5 (1–6.5)	0.267	*0.005*	0.2
Eosinophils	35 (24.5–45)	6 (5–7)	*< 0.001*	*< 0.001*	0.347
Mononuclear cells	30 (15–33.75)	21 (15–35)	0.745	*0.007*	*0.016*
Goblet cells	7.5 (5.25–13)	6 (5–135.5)	0.673	0.1	0.152
Mucosal gland	19 (7.5–28)	12 (7.5–15)	0.239	0.5	0.437
Inflammatory cells	73 (62–101)	33.5 (31.5–54)	*< 0.001*	*< 0.001*	0.152

Values are expressed as median (IQR). Mann-Whitney U test was used for unpaired comparisons

*CRSsNP* Chronic rhinosinusitis without nasal polyps, *ENP* Eosinophilic chronic rhinosinusitis with nasal polyps, *N-ENP* Non-eosinophilic chronic rhinosinusitis with nasal polyps

P < 0.05 was considered statistically significant. Italic P values show significant results (<0.05)

**Table 6:** Immunohistochemistry results in Eosinophilic and Non-eosinophilic CRSwNP subgroups

	ENP	N-ENP	ENP vs. N-ENP	ENP vs. CRSsNP	N-ENP vs. CRSsNP
Total IgA LP	11 (8.5–15)	5 (3–6)	*< 0.001*	*0.002*	*0.007*
IgA2 LP	2 (1–3)	0 (0–1)	*0.003*	*0.053*	0.11
IgA1 LP	10 (7–12.5)	4 (3–5)	*< 0.001*	*0.023*	*0.002*
Total IgA EP	3 (1.5–3)	1 (1–2)	*0.013*	*0.037*	0.99
IgA2 EP	1 (0–1)	0 (0–0)	*0.015*	0.123	0.47
IgA1 EP	2 (1–2)	1 (1–2)	0.179	0.85	0.611
B cells (CD20)	21 (8–44)	9 (7–16)	0.051	*0.026*	0.225
Plasma cells (CD138)	25 (7–42)	6 (4–14)	*0.022*	*0.01*	0.205

Values are expressed as median (IQR). Mann-Whitney U test was used for unpaired comparisons

*CRSsNP* Chronic rhinosinusitis without nasal polyps, *ENP* Eosinophilic chronic rhinosinusitis with nasal polyps, *N-ENP* Non-eosinophilic chronic rhinosinusitis with nasal polyps, *LP* Lamina propria, *EP* Epithelium

P < 0.05 was considered statistically significant. Italics P values show significant results (<0.05)

### BAFF, CXCL13, and GLTs were increased in CRSwNP patients

**Fig. 3:**
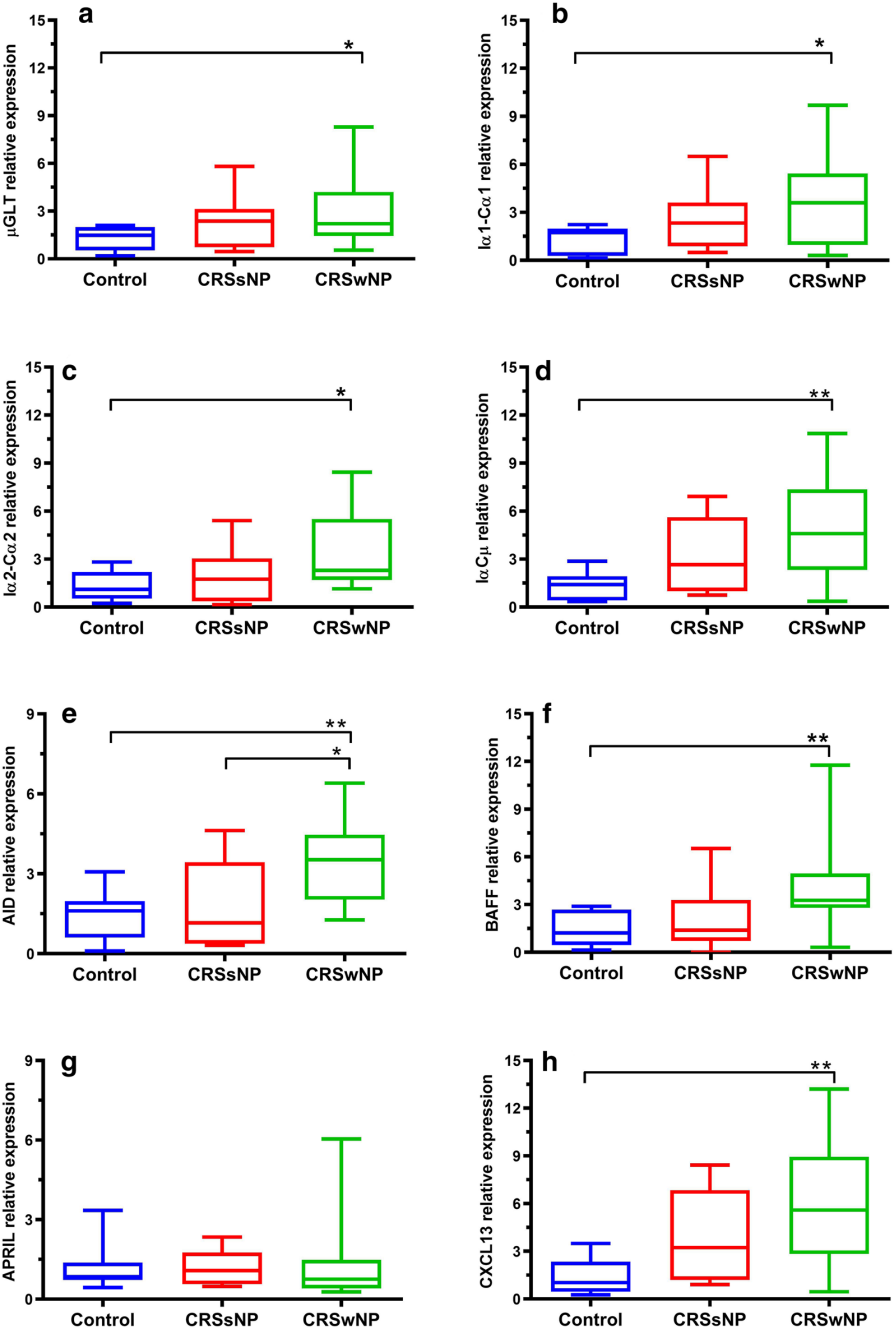
The mRNA expression levels of BAFF, Iα1-Cα1 GLT, Iα2-Cα2 GLT, Iα-Cµ CT, µGLT, APRIL, CXCL13, and AID in CRSwNP, CRSsNP, and control groups. The data are represented by box and whisker plots, and the Kruskal–Wallis test was used for the statistical analyses. *P < 0.05; **P < 0.01 *CRSsNP* chronic rhinosinusitis without nasal polyps, *CRSwNP* Chronic Rhinosinusitis with Nasal Polyps

The expression level of BAFF, IαCµ, and CXCL13 was significantly increased in CRSwNP patients compared with controls (*Pv* < 0.01) and no significant difference was observed between CRSsNP and CRSwNP groups (Fig. 3). It was also revealed that mRNA expression of Iα1Cα1, Iα2Cα2, and µGLT was significantly elevated in CRSwNP patients (*Pv* < 0.05) and no significant difference was observed between CRSsNP and CRSwNP groups (Fig. 3). The expression level of AID was significantly different between CRSwNP patients and both CRSsNP patients (*Pv* < 0.05) and controls (*Pv* < 0.01). Conversely, no difference was observed in terms of APRIL expression among the three groups (Fig. 3).

### Total IgA and IgA1 were increased in CRSwNP patients

**Fig. 4:**
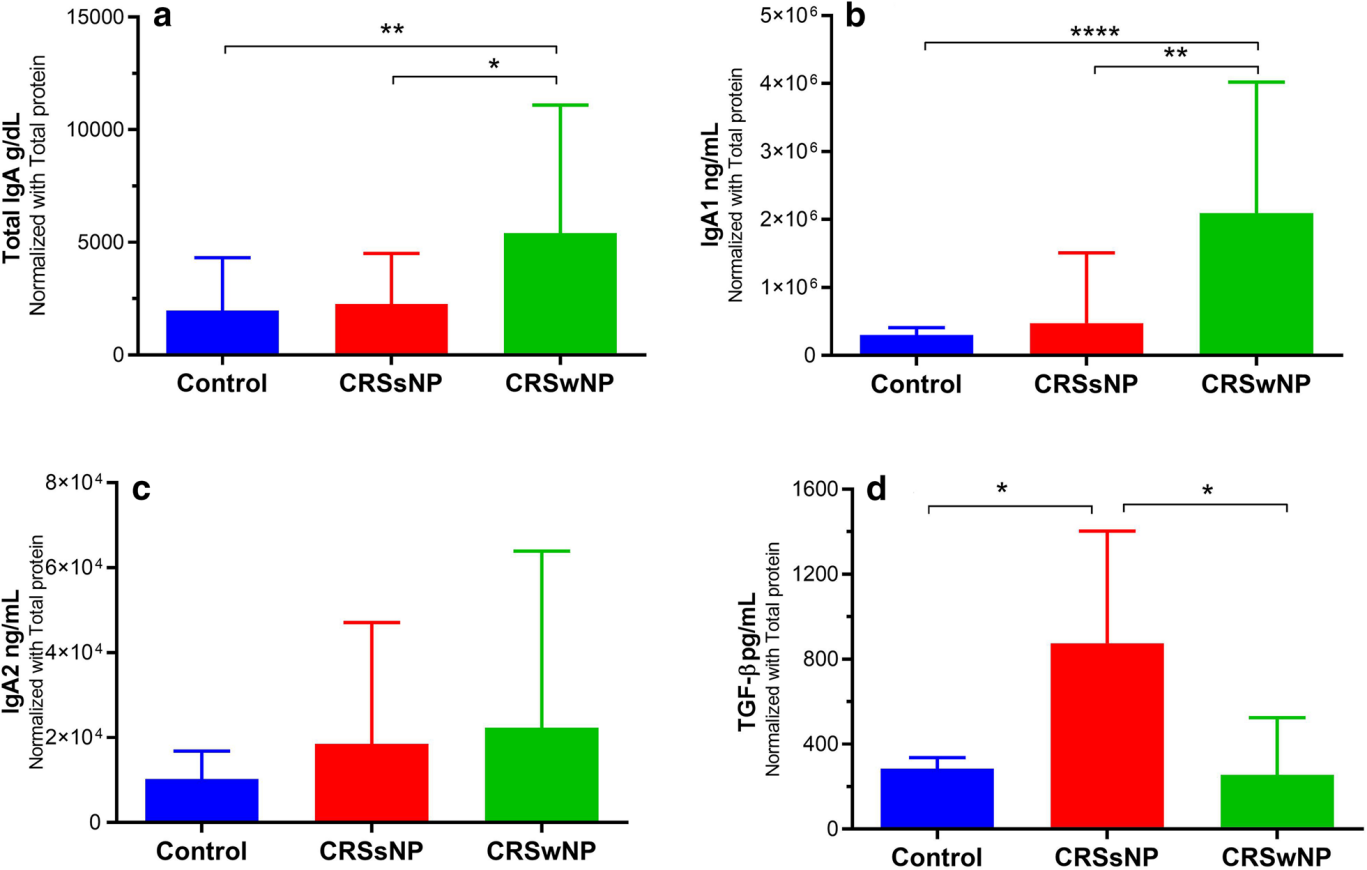
The protein levels of total IgA, IgA1, IgA2, and TGF-β in sinonasal tissue homogenates of CRSwNP, CRSsNP, and control groups. The concentrations of total IgA and IgA1 in tissue homogenates of CRSwNP patients were significantly increased in comparison to CRSsNP and control groups. There is no difference in the protein level of IgA2 in three groups. The data are represented by column and the Kruskal–Wallis test was used for the statistical analyses. *P < 0.05; **P < 0.01; ****P < 0.0001 *CRSsNP* chronic rhinosinusitis without nasal polyps, *CRSwNP* chronic rhinosinusitis with nasal polyps

**Fig. 5:**
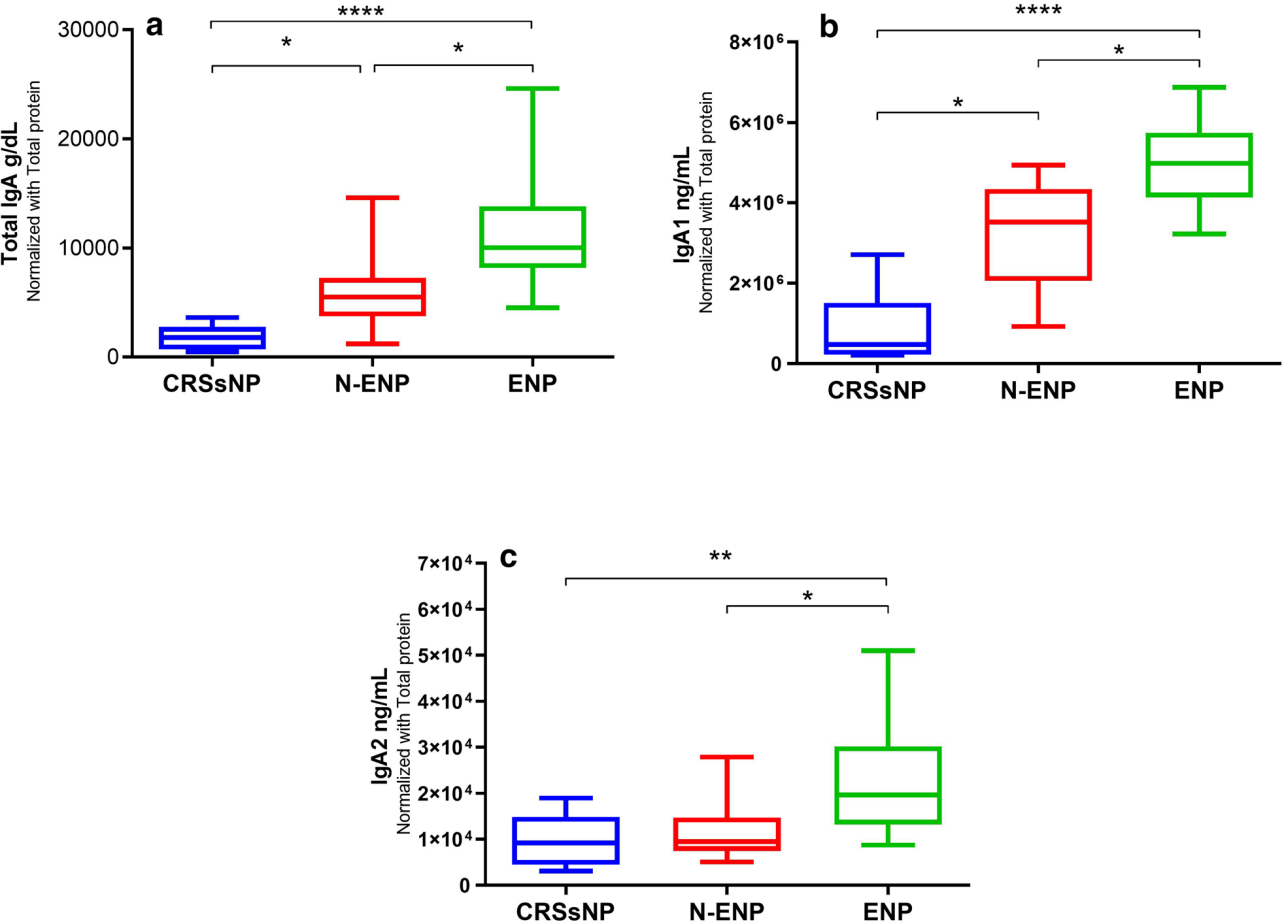
The protein levels of total IgA, IgA1, and IgA2 in ENP and N-ENP patients in comparison to CRSsNP group. The levels of total IgA and IgA1 in tissue homogenates of ENP patients were significantly increased in comparison to CRSsNP group. The level of IgA1 was increased in N-ENP rather than CRSsNP group. There is no difference in the protein level of IgA2 in three groups. The data are represented by column and bar plots and the Kruskal–Wallis test was used for the statistical analyses. *P < 0.05; **P < 0.01; ****P < 0.0001 *CRSsNP* Chronic rhinosinusitis without nasal polyps, *CRSwNP* Chronic rhinosinusitis with nasal polyps, *ENP* Eosinophilic chronic rhinosinusitis with nasal polyps, *N-ENP* Non-eosinophilic chronic rhinosinusitis with nasal polyps

The protein level of total IgA and IgA1 were significantly higher in CRSwNP patients than in other groups (Fig. 4). However, no difference was found in the IgA2 level of the studied groups (Fig. 4). The concentration of TGF-β was significantly increased in CRSsNP patients (Figure 4). Furthermore, the level of total IgA, IgA1 and IgA2 in ENP group were significantly higher than N-ENP group (Fig. 5).

## Discussion

Since the definitive mechanisms underlying IgA class switching in CRS patients are not completely understood, we evaluated the levels of total IgA and IgA subclasses as well as the involved mechanisms of IgA1 and IgA2 isotype switching in CRS patients and controls. Indeed, we evaluated inflammatory patterns of CRS patients and the protein levels of total IgA and IgA subclasses in addition to the expression levels of APRIL, BAFF, Iα1-Cα1GLT, Iα2-Cα2 GLT, μGLT, Iα–Cµ CT, AID, and CXCL13.

CRSwNP patients exhibited dominant eosinophilic inflammation in western populations but Chinese nasal polyps mostly exhibited non-eosinophilic inflammation [2]. Our results showed that 64% of CRSwNP patients were eosinophilic (ENP) and 36% were non-eosinophilic (N-ENP). Luo et al. showed that eosinophil infiltration had a direct correlation with disease severity and a higher CT and endoscopic scores. Their results suggested that eosinophilic status is associated with poor prognosis and long disease duration [22]. It has been demonstrated that eosinophils play pivotal roles in the pathogenesis of CRS. Eosinophils regulate the local inflammation in the sinonasal tissues of CRS patients [2]. Studies on eosinophil-deficient mice have demonstrated the important interactions of eosinophils and IgA expressing B cells. Hence, eosinophils can improve the development of IgA producing plasma cells locating in the LP [23].

In this study, total IgA and both subclasses were significantly elevated in LP of both CRS groups in comparison to controls whereas total IgA and IgA1 were significantly increased in the epithelium of only CRSwNP patients. Our findings were consistent with Van Zele et al. and Schleimer et al. study who showed that the concentration of IgA was higher in CRSwNP than the other two groups [24, 25]. We also evaluated the IgA and related subclasses in ENP and N-ENP patients. Total IgA and subclasses were significantly elevated in LP and epithelium (except IgA1) of ENP patients in comparison to N-ENP patients. These findings confirmed the Song et al. results in which IgA level in polyp with ectopic lymphoid tissues was increased in both ENP and N-ENP, but they did not evaluate IgA subclasses. Hupin et al. showed the level of IgA1 and IgA2 mature transcripts were not significantly different among studied groups [26]. Hammarstrőm et al. explored nasal mucosa of controls and indicated a continuous IgA1 class switching. They acclaimed that IgA1^+^ B cells could play a role as precursors of IgA2^+^ B cells and according to the content and type of the encountered antigens in the sinonasal mucosa, the proportion of IgA1^+^ and IgA2^+^ plasma cells could alter among various subjects [15]. We demonstrated that the expression of α1GLT was higher than α2GLT in CRSwNP patients. This finding may indicate that the transcription of IgA1 was higher than IgA2. Further investigation showed on the contrary to IgA2, the protein levels of total IgA and IgA1 were elevated in CRSwNP patients. In Kett et al. study, 96% of IgA^+^ cells were IgA1 positive in nasal mucosa [27]. According to our results, it sounds that the distribution rate of IgA1 and IgA2 doesn’t show alteration in nasal polyps; instead, their production is increased in comparison to normal tissues. In agreement with Dilidaer et al. study, our results show that the expression of AID mRNA was elevated in CRSwNP patients in comparison to controls [28]. In this study, the expressions of µGLT, Iα1-Cα1 GLT, Iα2-Cα2 GLT, Iα–Cµ CT, and AID showed an ongoing IgA subclass switching in CRSwNP patients.

Interestingly, we found a positive correlation between eosinophil counts and the levels of IgA subclasses in CRSwNP patients. Human eosinophils express various IgA receptors, including FcαRI (CD89), poly Ig receptor (pIgR) and so on [29]. Elevated FcαRI expression has been frequently reported on eosinophils of allergic and asthmatic patients and impaired expression of FcαRI may result in enhanced IgA levels [30]. Altogether, further research is necessary to determine the functions of receptors related to IgA subclasses on eosinophils.

Class switching is regulated by a number of cytokines. TGF-β1 is a prominent cytokine, providing IgA switching through the regulation of germline transcripts [15]. In our study, although the level of TGF-β in CRSwNP patients decreased in comparison to the control group, which was not significant, this difference was significant in Bruaene et al. study [31]. Responsive elements of TGF-β1 in the human Iα1 and Iα2 promoters have a similar structure, so this cytokine probably does not determine the direction toward IgA subclasses [32]. Hence, other prerequisite factors are needed. BAFF is a potent cytokine to enhance the induction of IgA1 and IgA2 expression and APRIL can only stimulate IgA2 expression [11]. It has been reported that BAFF is elevated in CRSwNP patients, thus it may be a hallmark of active IgA class switching [33, 34]. In our study, the expression of BAFF was significantly elevated in CRSwNP patients compared with controls; while the expression of APRIL didn’t show any significant difference in three groups. This is consistent with the study of Schleimer et al. who reported that BAFF mRNA may be significantly elevated in CRSwNP patients. Meanwhile, they showed that the expression of APRIL wasn’t increased in CRS patients [24]. Yon et al. showed that BAFF^+^ cells were higher in eosinophilic CRSwNP group than non-eosinophilic and controls. Their study also showed BAFF expression was significantly correlated with IgA production and B cell accumulation [35]. He et al. reported that the expression of APRIL in intestinal epithelial cells is higher than other epithelial cells. It may be due to the inducers of APRIL expression, including lipopolysaccharide (LPS) and flagellin which are commonly found in the intestine where they can stimulate TLR4 and TLR5, respectively [11]. However, LPS and flagellin do not alter the expression of APRIL in human nasal epithelial cells which predominantly express TLR2 and TLR3 instead [24]. Indeed, the main difference between BAFF and APRIL expression is defined by the final purpose of IgA class switching whether it is protective against commensal or infectious microbes [24].

CXCL13 is an important factor that recruits B cells to the local inflammatory sites and it has been previously established to be elevated in CRSwNP patients [36]. IHC results indicated CD20^+^ cells and CD138^+^ cells were increased in CRSwNP patients which were consistent with Dilidaer et al. study [28]. We found that the expression of CXCL13 was significantly elevated in CRSwNP patients. On combination of increased number of B cells and the level of IgA in CRSwNP patients, it is possible that CXCL13 actively contributes to the local IgA class switching due to the recruitment of B cells to the inflammatory sites. Cao et al. indicated that the expression of CXCL13 is increased in both ENP and N-ENP patients. They also showed that the number of CD20^+^ and CD138^+^ cells is increased in both groups [37]. Future studies are needed to reveal how these chemokines facilitate the recruitment of B cells to the inflammatory sites of the nasal mucosa.

Our findings provided new evidence suggesting possible mechanisms in IgA subclass switching and correlation with eosinophil in CRSwNP patients. However, more studies are needed to validate the exact mechanisms in association with IgA and eosinophil activation in these patients. Furthermore, we could not verify the relationship between IgA class switching process and eosinophil in CRSsNP patients; Therefore, further investigations are necessary to clarify the impacts of IgA in CRSsNP patients. Further studies in this area may reveal unknown mechanisms in patients and can also reveal new therapeutic goals. The major limitation of this study was small the sample population.

## Conclusion

In the current study, the factors involved in the promotion of IgA class switching in the sinonasal mucosa of CRS patients were evaluated. These findings showed that IgA subclasses were increased in the CRSwNP patients. We also showed that eosinophils correlated with IgA positive cells in the CRSwNP patients. Furthermore, BAFF, CXCL13, GLTs, and AID were significantly increased in the CRSwNP patients possibly demonstrating mechanisms of IgA subclass switching in CRS patients. Moreover, the findings showed IgA subclasses in the ENP group were significantly higher than the N-ENP group. In future studies, more attention should be given to the relationship of IgA receptors and its signaling pathway with eosinophilic inflammation. In this regard, the study of whether the recruitment of eosinophils should be done by IgA or vice versa. Further studies on the role of local production of IgA and its relationship with pathologic functions of eosinophils may lead to designing novel inhibitors to reduce the inflammation and the ENP patient’s symptoms.

## Data Availability

Not applicable.
